# Defining resistance and tolerance traits in Covid-19: towards a stratified medicine approach

**DOI:** 10.1093/qjmed/hcac143

**Published:** 2022-06-10

**Authors:** C D Russell, S Clohisey Hendry

**Affiliations:** Queen’s Medical Research Institute, University of Edinburgh Centre for Inflammation Research, Edinburgh BioQuarter, 47 Little France Crescent, Edinburgh EH16 4TJ, UK; Division of Genetics and Genomics, Roslin Institute, University of Edinburgh, Easter Bush Campus, Midlothian EH25 9RG, UK

## Abstract

Successful host defence against infectious disease involves resistance (reduce pathogen load) and tolerance (reduce tissue damage associated with pathogen presence). Integration of clinical, immunologic, genetic and therapeutic discoveries has identified defects in both of these responses in the progression from SARS-CoV-2 infection to life-threatening coronavirus disease 2019 (Covid-19) lung injury. Early after infection with SARS-CoV-2, resistance can be compromised by a failed type 1 interferon (IFN-I) response, due to direct viral antagonism of induction and signalling, deleterious host genetic variants (*IFNAR2, IFNA10, TYK2* and *PLSCR1*), and neutralizing auto-antibodies directed against IFN-I (predominantly IFN-α). Later in the disease, after pathogen sensing has activated a pro-inflammatory response, a failure to appropriately regulate this response compromises tolerance resulting in virus-independent immunopathology involving the lung and reticuloendothelial system. Monocytes are activated in the periphery (involving M-CSF, GM-CSF, IL-6, NLRP1 inflammasomes, TYK2 and afucosylated anti-spike IgG) then recruited to the lung (involving CCR2::MCP-3/MCP-1 and C5a::C5aR1 axes) as pro-inflammatory monocyte-derived macrophages, resulting in inflammatory lung injury. Phenotypic and genotypic heterogeneity is apparent in all these responses, identifying ‘treatable traits’ (therapeutically relevant components of inter-individual variation) which could be exploited to achieve a stratified medicine approach to Covid-19. Overall, Covid-19 pathogenesis re-affirms the importance of resistance in surviving an infectious disease and highlights that tolerance is also a central pillar of host defence in humans and can be beneficially modified using host-directed therapies.

## Introduction

Host defence against infectious disease comprises responses to affect resistance (reduce pathogen load) and the more recently recognized concept of tolerance (reduce tissue damage associated with pathogen presence).^1^ Therapeutically augmenting resistance is familiar to clinicians through using antimicrobial drugs. Augmenting tolerance is comparatively uncommon in clinical practice and restricted to specific scenarios, for example, anti-inflammatory corticosteroids to treat tuberculosis-associated immune reconstitution inflammatory syndrome. Prior to the discovery of effective therapies, the in-hospital mortality for people with coronavirus disease 2019 (Covid-19) at the start of the pandemic exceeded 30%.^2^ Death in Covid-19 occurs due to hypoxic respiratory failure caused by inflammatory lung injury. Integration of clinical, immunologic, genetic and therapeutic discoveries identifies defects in both resistance to and tolerance of SARS-CoV-2 infection in progression to life-threatening disease. Heterogeneity is apparent in clinical features,^3^ host immune responses^4^ and genetic susceptibility^5^ to Covid-19, and sub-groups of patients with mechanistic differences in the biology of their disease exist. Components of this inter-individual variation in disease pathophysiology that determine differential responses to therapeutic interventions (‘treatable traits’)^6^ could identify additional therapeutic targets and contribute to a stratified medicine approach to Covid-19. In this article, we summarize the roles of resistance and tolerance in Covid-19 pathogenesis, focusing on innate immunity and highlight relevant treatable traits.

## Resistance: defective type 1 interferon responses

**Figure 1.**
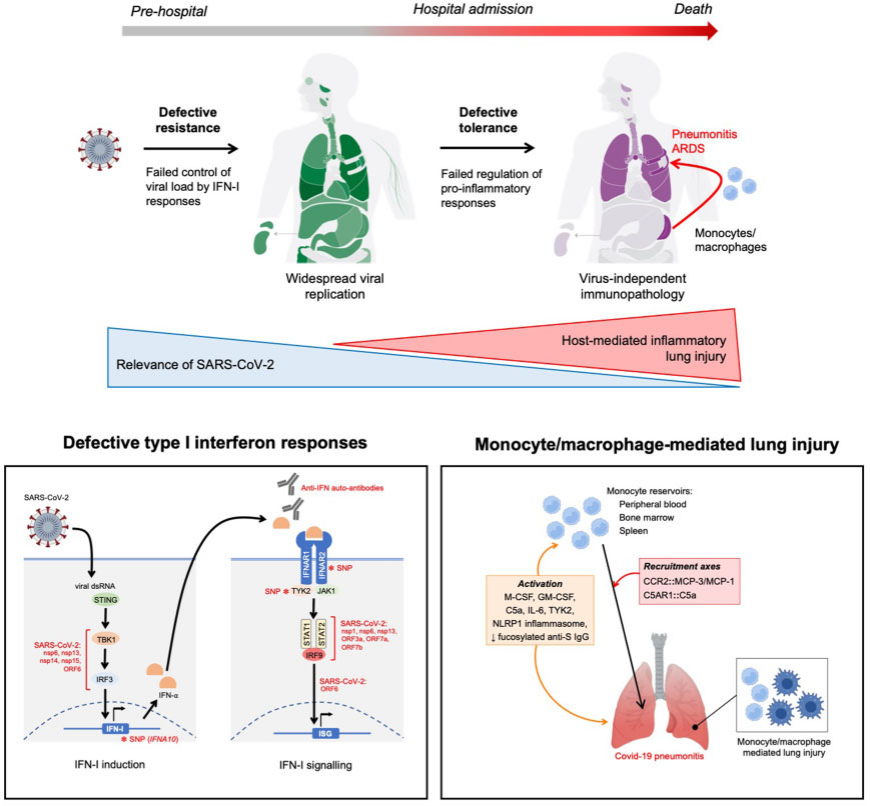
Resistance and tolerance in life-threatening Covid-19. Defects in resistance and tolerance compromise host defence at distinct stages of Covid-19 pathogenesis. Early after initial infection with SARS-CoV-2, resistance can be compromised by a failed type 1 interferon (IFN-I) response, due to direct viral antagonism, host genetic variants and auto-antibodies directed against IFN-I (inset box 1). SARS-CoV-2 genes associated with disruption of host IFN-I signalling: nsp6, nsp13, nsp14, nsp15, ORF6, ORF3a, ORF7a and ORF7b. Intensity of shading in the anatomic diagram represents the frequency of detection of viral RNA by multiplex PCR and sequencing in a post-mortem study of 11 people with fatal Covid-19.^7^ Later in disease, after pathogen sensing has activated a pro-inflammatory response, a failure to appropriately regulate this response compromises tolerance and results in immunopathology. Intensity of purple shading in the anatomic diagram represents the results of semi-quantitative scoring of histologic evidence of inflammation in the same post-mortem study, disconnected from the widespread presence of virus.^7^ The end result of this is monocyte/macrophage-mediated inflammatory lung injury, after activation and mobilization of monocytes in the periphery and recruitment to the lung as pro-inflammatory monocyte-derived macrophages (inset box 2). The anatomic diagrams are modified from reference 7. Created with BioRender.com.

Type 1 interferon (IFN-I) signalling is a canonical innate immune response mediating resistance during viral infections. Orthogonal evidence identifies a critical role for IFN-I responses in host resistance to SARS-CoV-2 and implicates a failure of these responses in life-threatening diseases (Figure 1). SARS-CoV-2 inhibits distinct elements of the IFN-I response *in vitro*. IFN-I production is reduced by interference with the transcription factor IRF-3, which usually mediates IFN-α/β induction in response to intracellular viral dsRNA.^8–10^ Processes downstream of IFN-I receptor (IFNAR) activation are also targeted, resulting in the impaired formation and reduced nuclear translocation of the STAT1/STAT2/IRF9 complex that usually leads to IFN-stimulated gene (ISG) induction.^8^^,^^10^ In people hospitalized with Covid-19, IFN-α concentrations in blood are substantially lower than in people with H1N1/H3N2 influenza A virus disease, a finding consistent with *in vitro* observations in infected airway epithelia.^11^^,^^12^ Compared to mild/moderately severe Covid-19, IFN-α concentrations are lower in critically ill people, and in people progressing to require invasive mechanical ventilation (IMV), suggesting this failure is associated with disease severity.^13^^,^^14^ Circulating IFN-α is undetectable in a sub-set of critically ill people with Covid-19 (unrelated to illness duration). The presence of neutralizing auto-antibodies directed against IFN-I (predominantly IFN-α) is associated with life-threatening Covid-19, but not asymptomatic/mild SARS-CoV-2 infection and is more common in both males and people aged ≥65 years.^15^*In vitro*, plasma from patients with these auto-antibodies abolishes IFN-I signalling in peripheral blood mononuclear cells and prevents IFN-α restriction of SARS-CoV-2 infection in Huh7.5 cells. The presence of auto-antibodies is associated with reduced ISG expression in both blood and the nasopharynx.^16^

Susceptibility to infectious disease is a strongly heritable trait and findings from genome-wide association studies (GWAS) implicate risk alleles associated with a defective IFN response in critical illness due to Covid-19.^5^^,^^17^ Potential consequences of these risk alleles include reduced *IFNAR2* expression, a destabilizing amino acid substitution in IFN-α10 (*IFNA10*), reduced STAT1 phosphorylation by TYK2 and disruption of phospholipid scramblase 1 (*PLSCR1*) nuclear localization (which is causally associated with ISG expression).^17^ Although direct evidence that defective IFN-I responses are associated with higher viral loads is lacking, it has been demonstrated that defective host resistance (i.e. increased SARS-CoV-2 viral load in the respiratory tract and peripheral blood) is associated with both the severity of the respiratory failure and mortality.^18–20^

## Tolerance: monocyte/macrophage-mediated inflammatory lung injury

Anti-inflammatory therapy with corticosteroids and anti-IL-6 receptor monoclonal antibodies reduces mortality in hospitalized people with respiratory failure due to Covid-19, indicating inflammation is causal in death in the later stages of the disease.^21^^,^^22^ Post-mortem investigation of tissue from people who have died due to Covid-19 identifies a mismatch between the distribution of SARS-CoV-2 and histologic evidence of inflammation.^7^^,^^23^^,^^24^ At the time of death, viral RNA and spike glycoprotein are present in a wide range of tissues. However, the inflammatory response is concentrated in the lungs, spleen and bone marrow (Figure 1). In other organs with evidence of viral presence, inflammatory responses are not consistently present. Even within the lung of individual patients, inflammation does not map consistently with the distribution of the virus: inflammation can occur without the viral presence and the virus can be present but without an associated inflammatory response.^7^ This indicates that inflammation in fatal Covid-19 is a virus-independent immunopathologic process. This failure to appropriately regulate pro-inflammatory responses downstream of pathogen sensing represents defective tolerance in late disease and appears to be specific to the lung and reticuloendothelial environments (Figure 1).

An expansion of activated and proliferating monocytes is observed in peripheral blood and bronchoalveolar lavage fluid in Covid-19, accompanied by a selective expansion of monocytes/macrophages in the lung parenchyma in fatal disease.^7^^,^^25–27^ These are predominantly interstitial (monocyte-derived) macrophages (MDM) recruited from peripheral blood,^26^ and their expansion in Covid-19 pneumonitis is quantitatively greater than that observed in influenza and bacterial pneumonia, identifying a specific role for these cells in Covid-19 immunopathogenesis.^7^^,^^25^ Both MDM and alveolar macrophages in Covid-19 lung tissue display aberrantly activated transcriptional phenotypes.^26^ Macrophages are associated with pulmonary vascular inflammation, with findings of obliterative endarteritis and pulmonary artery vasculitis involving monocytes/macrophages.^7^^,^^28^ Abnormal macrophage phenotypes are also present in the bone marrow, involving iron loading and haemophagocytosis. In a humanized mouse model of Covid-19 (MISTRG6 mice expressing hACE2), SARS-CoV-2 infection is associated with an expansion of macrophages in the lung.^29^ Dexamethasone treatment prevents infection-associated weight loss and is associated with a reduction in inflammatory macrophages in the lung,^29^ providing evidence these cells are causal in pathogenesis and suggesting a mechanism of action of dexamethasone. Despite evidence of an activated hyper-inflammatory neutrophil phenotype in peripheral blood in Covid-19,^30^ and contribution of neutrophil extracellular traps to pathogenesis,^31^ there is comparatively less evidence of a major role of neutrophils as effectors of lung injury.^7^^,^^25^ This pathological role of monocytes/macrophages is a departure from their classical role in host defence against pathogens, where they usually contribute to pauci-inflammatory microbicidal responses then tissue repair and regeneration.

Potential mechanisms mediating dysregulated monocyte/macrophage responses have been defined. The myeloid cell growth factors M-CSF (CSF-1) and GM-CSF (CSF-2) contribute to monocyte activation and recruitment to sites of inflammation.^32^^,^^33^ Circulating M-CSF is elevated in moderate/severe disease and concentrations are also increased in the spleen in fatal Covid-19.^34^^,^^35^ Splenic monocytes can be deployed to distal sites of inflammation so may contribute to pulmonary MDM infiltration in Covid-19. A specific role for GM-CSF (CSF-2) has also been identified, with concentrations in the blood increasing with disease severity, in contrast to influenza where no elevation is observed.^4^ Expression of the GM-CSF receptor (*CSF2RA*) is up-regulated on MDM in lung tissue.^26^ DPP9, which is implicated in COVID-19 GWAS, is a negative regulator of NLRP1/CARD8 inflammasomes and functional loss is associated with increased IL-1β and IL-18 secretion, both of which are associated with Covid-19 severity.^4^^,^^36^ Finally, a genetic variant associated with increased *TYK2* expression (and reduced STAT1 phosphorylation) is also associated with a critical illness.^5^ The Janus Kinase TYK2 is involved in IL-1β and IL-18 secretion by macrophages.^37^

A transcriptome-wide association study showed increased expression of *CCR2* (encoding C-C Motif Chemokine Receptor 2) in lung tissue was associated with critical illness in Covid-19. CCR2 is involved in monocyte/macrophage recruitment to sites of inflammation and its two ligands, MCP-3 and MCP-1, are both implicated in pathogenesis. MCP-3 is elevated in inflamed lung tissue and blood in fatal^35^ and severe Covid-19, respectively, whereas circulating MCP-3 is not elevated in influenza.^34^^,^^38^ Circulating MCP-1 concentrations and MCP-1 expression by bronchoalveolar lavage fluid macrophages also both scale with disease severity.^4^^,^^39^ Circulating monocytes express C5aR1 and binding of its ligand, the complement component C5a, mediates activation and recruitment to sites of inflammation. In Covid-19, circulating C5a concentrations are associated with disease severity, C5a is detectable in BALF, and macrophages infiltrating the lung parenchyma express C5aR1 (including macrophages associated with vasculitis and micro-thrombosis).^28^ In addition, C5a augments CCL2 and IL-6 production from monocytes from people with Covid-19 after *ex vivo* LPS stimulation.^28^ Overall, there is evidence for monocyte activation in the periphery, involving M-CSF, GM-CSF, DPP-9 and C5a; then recruitment to the lung involving CCR2::MCP-3/MCP-1 and C5a::C5aR1 axes.

The adaptive IgG response to SARS-CoV-2 can also negatively impact on tolerance. Afucosylation of the Fc of IgG is associated with increased affinity for the FcγRIIa and enhanced activation of myeloid phagocytes. Anti-spike protein IgG1 Fc fucosylation is variable in critically ill people with ARDS due to Covid-19 but is overall reduced in comparison to asymptomatic or mildly ill people.^40^ Afucosylated IgG is also more common in men.^41^ Anti-S IgG1 Fc fucosylation is negatively correlated with circulating IL-6 and CRP, and these mediators increase in concentration at the same time as afucosylated anti-S IgG is first produced.^40^*In vitro*, anti-S IgG with reduced fucosylation (from Covid-19 patients) and a recombinant anti-S monoclonal antibody with reduced fucosylation stimulate macrophage production of pro-inflammatory mediators including IL-6 and IL-8.^41^^,^^42^

## Treatable traits in Covid-19

### IFN-I responses

Clinical trials investigating IFN therapy as an approach to augment host resistance have been disappointing. Trials of IFN-β for hospitalized people have demonstrated no benefit,^43^ and in one trial outcomes were worse in people with more severe respiratory failure.^44^ This could suggest that IFN therapy should be administered earlier in the disease. Although IFN-β has not been investigated by randomized controlled trial in this context, two small trials of type III IFN (IFN-λ) in outpatients (where treatment was started 4.5–5 days after illness onset) did not demonstrate a convincing benefit.^45^^,^^46^ The disconnect between the importance of IFN-I in host defence and negative clinical trial results highlights an unmet need in patient stratification that embraces the heterogeneity observed in IFN-I responses. It is plausible that only a specific endotype of patient will benefit, hypothetically: (1) elevated viral load in nasopharynx or blood; (2) evidence of failed induction of an IFN-I response (e.g. due to auto-antibodies against IFN-α or risk allele for *IFNA10*); and (3) intact IFN signalling (no risk allele for *IFNAR2* or *PLSCR1*). Although auto-antibodies abrogate endogenous IFN-I responses, given the rarity of anti-IFN-β antibodies (in contrast to anti-IFN-α) these people may still benefit from IFN-β therapy. There is encouraging evidence that resistance can be augmented early after infection to achieve the clinical benefit, evidenced by the reduction in hospitalization with outpatient administration of anti-viral drugs (nirmatrelvir plus ritonavir and remdesivir) and neutralizing monoclonal antibodies early after symptom onset. In addition to stratification by clinical risk factors (e.g. co-morbidities), evidence of defective IFN-I responses could also be used to stratify the outpatient usage of these therapies.

### Monocyte/macrophage responses

A specific role for pro-inflammatory MDM has been demonstrated in Covid-19. Genetic variation associated with differential *CCR2* or *TYK2* expression, combined with stratification using circulating GM-CSF or C5a, could identify endotypes expected to differentially respond to specific therapies. Anti-CCR2 monoclonal antibody therapy (MLN1202) has been investigated safely in people with rheumatoid arthritis, where it reduced monocyte counts and free CCR2 on circulating monocytes.^47^ Results of a small clinical trial of anti-GM-CSF monoclonal antibody therapy (otilimab) in Covid-19 found no benefit overall, but in people aged ≥70 years, outcomes were improved.^48^ This pre-planned sub-group analysis was based on the observation that when circulating GM-CSF concentrations are stratified by this age threshold, GM-CSF is substantially higher in people aged ≥70; providing important proof of principle of the utility of biologically informed patient stratification.^4^ In people with elevated C5a, blockade of the receptor could improve outcomes. Avdoralimab is an anti-C5aR1 monoclonal antibody that can prevent C5a-induced acute lung injury in mice and specifically block monocyte recruitment to the lung.^28^ This approach has been evaluated in a clinical trial in Covid-19 (NCT04371367), but the results have not yet been reported. Finally, stratification of JAK inhibitor therapy with baricitinib, which also has TYK2 inhibitory activity, could be stratified by *TYK2* genotype or expression to identify people likely to derive maximal benefit.^49^^,^^50^

### Pro-inflammatory afucosylated IgG

Clinical deterioration associated with hospitalization and requirement for IMV often occurs in the second week of illness in Covid-19, temporally associated with IgG seroconversion (which is quantitatively greater in more severe diseases).^51^ The finding that a sub-group of patients with severe disease have abnormally reduced fucosylation of anti-S IgG Fc, associated with pro-inflammatory macrophage responses, identifies this as a treatable trait. FcγR signalling requires the Syk kinase and *in vitro*, inhibition with the small molecular inhibitor fostamatinib suppressed anti-S IgG induced Il-6, IL-8 and IL1β secretion by macrophages.^42^ A small clinical trial suggests fostamatinib may be beneficial in hospitalized people,^52^ but stratification by IgG glycosylation status could identify a sub-group of patients likely to derive greater benefit.

## Conclusions

**Figure 2.**
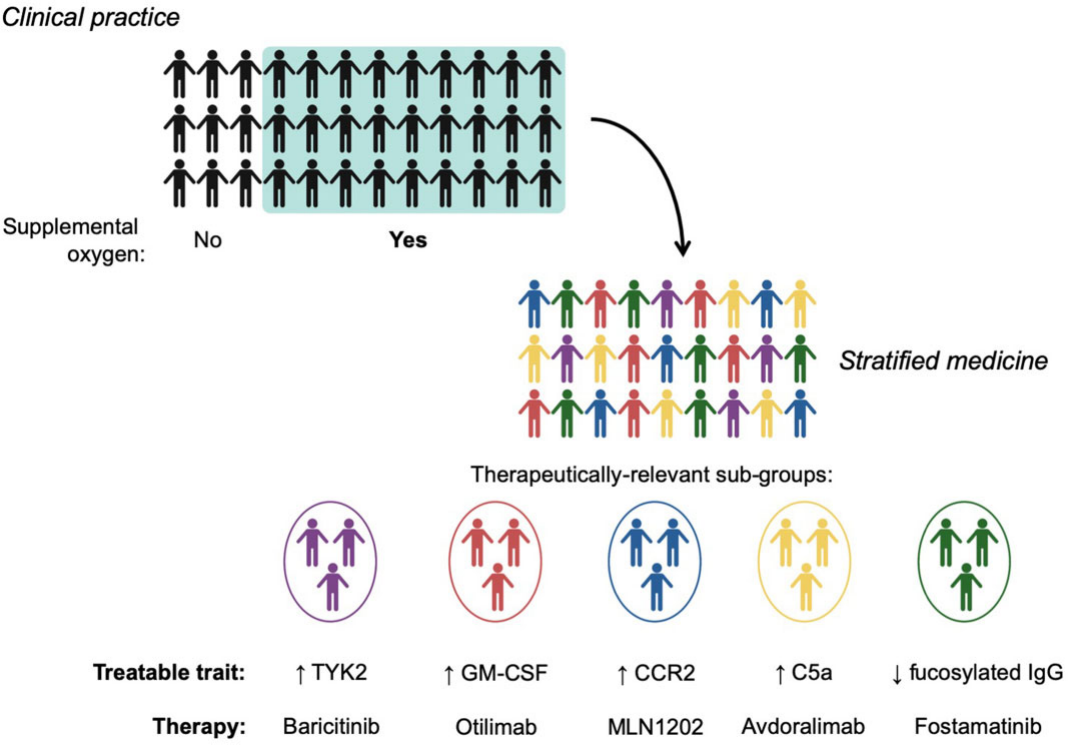
Treatable traits in Covid-19 inform a stratified medicine approach. Clinical trial data demonstrate that dexamethasone only reduces mortality in hospitalized people with Covid-19 who require supplemental oxygen. In clinical practice, this degree of stratification is used routinely in the management of Covid-19 but people requiring supplemental oxygen still represent a biologically heterogeneous sub-group, composed of multiple disease endotypes (represented in different colours) that are likely to respond differently to more targeted immunomodulatory therapies. A stratified medicine approach to Covid-19 could see biologically informed stratification of these patients to identify therapeutically relevant sub-groups with shared treatable traits, identified using circulating mediators (GM-CSF, C5a), genotype (*TYK2*, *CCR2*) or anti-spike IgG Fc fucosylation status. Created with BioRender.com.

Covid-19 pathogenesis re-affirms the importance of resistance in surviving an infectious disease and highlights that tolerance is also a central tenet of host defence in humans and can be beneficially modified using host-directed therapies. The broad disease mechanism of life-threatening Covid-19 is homogeneous: inflammatory lung injury resulting in hypoxic respiratory failure, associated with large effect sizes in clinical trials of anti-inflammatory corticosteroids. However, GWAS, transcriptomics and proteomics have identified genotypic and phenotypic heterogeneity in immune responses in people with life-threatening Covid-19, in processes involved in both resistance and tolerance. Integrating these findings to exploit the treatable traits associated with this heterogeneity could further improve outcomes in severe diseases through a stratified medicine approach to Covid-19 (Figure 2).

## Funding

This work was supported by the Medical Research Council [grant number MC_PC_19059]. C.D.R. is supported by an Edinburgh Clinical Academic Track (ECAT)/Wellcome Trust PhD Training Fellowship for Clinicians award (214178/Z/18/Z). S.C.H. is funded by central BBSRC funding at The Roslin Institute.

*Conflict of interest*: None declared.
